# Association of ABO blood group, Rh phenotype and MN blood group with susceptibility to COVID-19

**DOI:** 10.1371/journal.pone.0296917

**Published:** 2024-01-19

**Authors:** Genjie Lu, Wei Chen, Yangfang Lu, Qilin Yu, Li Gao, Shijun Xin, Guanbao Zhou

**Affiliations:** 1 Department of Blood Transfusion, Ningbo Medical Center Lihuili Hospital, Ningbo University, Ningbo, China; 2 Department of Radiotherapy, Ningbo Medical Center Lihuili Hospital, Ningbo University, Ningbo, China; 3 Department of Hepatopancreatobiliary Surgery, The First Affiliated Hospital of Ningbo University, Ningbo, China; Roswell Park Cancer Institute, UNITED STATES

## Abstract

**Background:**

Previous studies have reported that the susceptibility to coronavirus disease 2019 (COVID-19) is related to ABO blood group, but the relationship with Rh phenotype and MN blood group is unknown. China had adopted a strict control policy on COVID-19 until December 5, 2022, when local communities were liberalized. Therefore, we aimed to explore the correlation between ABO blood group, Rh phenotype, MN blood group and susceptibility to COVID-19 based on the time sequence of infection during the pandemic.

**Methods:**

A total of 870 patients who were routinely hospitalized in Ningbo Medical Center Lihuili Hospital from March 1, 2023 to March 31, 2023 were randomly selected to enroll in this study. Patients were divided into susceptible group and non-susceptible group, according to the time of their previous infection. The demographics and clinical information of the enrolled participants were collected from electronic medical records. The association of ABO blood group, Rh phenotype and MN blood group with susceptibility to COVID-19 was analyzed.

**Results:**

A total of 650 cases (74.7%) had been infected with COVID-19, with 157 cases (18.0%) in the second week and 252 cases (29.0%) in the third week, reaching the peak of infection. Compared with the non-susceptible group, the susceptible group had no statistically significant differences in ABO blood group and Rh phenotype, but the proportion of N+ was higher (75.6% vs 68.9%, *P* = 0.030) and the proportion of MM was lower (24.4% vs 31.1%, *P* = 0.030). Consistent with this, ABO blood group and Rh phenotype were not significantly associated with susceptibility to COVID-19 (*P>*0.05), while N+ and MM were associated with susceptibility to COVID-19 (OR: 1.432, 95% confidence interval [CI]: 1.049, 1.954, *P* = 0.024; OR: 0.698, 95% CI: 0.512, 0.953, *P* = 0.024, respectively), after adjusting for age, sex, BMI, basic disease, and vaccination status in multivariate logistic regression analysis.

**Conclusion:**

Our study showed that ABO blood group and Rh phenotype may not be related to the susceptibility to COVID-19, but MN blood group may be associated with the susceptibility to COVID-19.

## Introduction

In December 2019, an unprecedented form of pneumonia appeared in Wuhan, China, and quickly spread around the world [1, 2]. The pneumonia was later confirmed to be caused by severe acute respiratory syndrome coronavirus 2 (SARS-CoV-2) [3], and was named coronavirus disease 2019 (COVID-19) by the World Health Organization.

There are many factors affecting susceptibility to COVID-19, including allergies and asthma [4], human leukocyte antigen (HLA) [5, 6], angiotensin converting enzyme (ACE) [7], ABO blood type [8–10], etc.

Previous studies have found that ABO blood groups are associated with susceptibility to a variety of infectious diseases [11–14]. In particular, the correlation between ABO blood groups and SARS-CoV has directly led to the hypothesis of similar susceptibility to COVID-19. Most studies so far have found that A blood group is more susceptible to COVID-19, while O blood group is less susceptible [15–17]. However, many other studies have not found such an association between ABO blood group and susceptibility to COVID-19 [18, 19]. Therefore, further studies are warranted.

RhD blood type has been reported to be associated with COVID-19 infection [17, 20, 21]. A recent study by Zietz et al. [17] found that RhD negative individuals are more likely to be infected with COVID-19 than RhD positive individuals. However, the Rh phenotype includes not only D antigen, but also C, c, E, e antigens. At present, it is unknown whether the Rh phenotype is related to susceptibility to COVID-19. At the same time, the correlation between MN blood group and COVID-19 infection has not been studied.

China had adopted a strict control policy on COVID-19 until December 5, 2022, when local communities were liberalized. Therefore, in order to better understand the correlation between ABO blood group, Rh phenotype, MN blood group and susceptibility to COVID-19 based on the time sequence of infection during the pandemic, a prospective cohort study was performed in Ningbo Medical Center Lihuili Hospital, Ningbo University, Ningbo, Zhejiang province, China.

## Materials and methods

### Date collection and study design

This was a prospective cohort study performed at Ningbo Medical Center Lihuili Hospital from March 1, 2023 to March 31, 2023. During this time, all interested patients were recruited to a physician’s office prior to the start of the study for notification and to obtain written informed consent from each enrolled participant. Among them, the minors obtained the written informed consent of their guardians.

**Fig 1:**
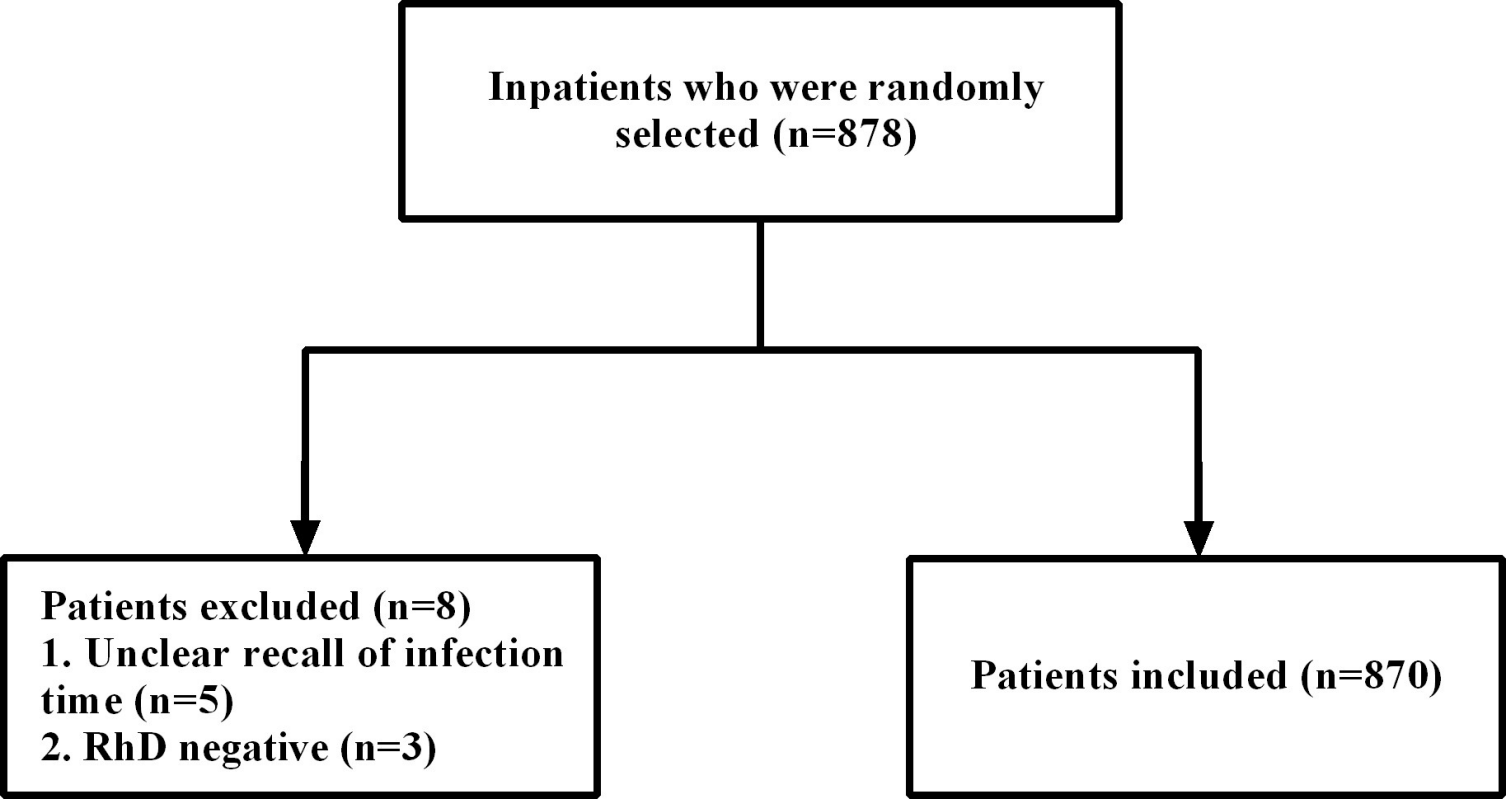
Consort diagram detailing the selection and exclusion of patients.

In this study, all routine inpatients were eligible for inclusion. 878 patients were randomly selected at first. After excluding 5 patients with unclear recall of infection time and 3 RhD negative patients, a total of 870 patients were enrolled in the study (Fig 1). At the same time, in order to minimize selection bias, 28,321 Rh (D) positive hospitalized patients from the same hospital prior to the onset of the local COVID-19 outbreak throughout 2019 were selected as controls.

Detailed time of previous COVID-19 infection from 5 December 2022 to the start of the study was obtained by asking patients or family members. In order to reduce recall bias, special days were used to help patients recall, including Christmas, New Year’s Day, Chinese New Year, etc. Patients were diagnosed mainly by self testing COVID-19 antigen or going to the hospital to test nucleic acid. The detailed number of vaccinations were obtained through Alipay software (Alipay (China) Network Technology Co., Ltd., China) on patient’s mobile phone or by asking the patient directly. The demographics and clinical information, including age, sex, BMI, basic disease (malignancy, hypertension, diabetes), were obtained by electronic medical records (EMR, KINGT software v5.0) (Ningbo Jintang Software Co., Ltd, China).

The remaining samples of the patient’s routine blood routine were used for testing, and no additional specimens were collected. ABO blood group detection and Rh phenotype detection were performed with HAMILTON automatic Blood Group instrument 185015 (HAMILTON, Switzerland). MN blood group was detected by manual test tube method with anti-M and anti-N serum reagents (Shanghai Blood Center, China). All operations were carried out in strict accordance with the standard operating procedures, and the indoor quality control was in control during the effective period of the reagent.

Previously, our country had adopted a strict control policy against COVID-19 until December 5, 2022, when the local community liberalized. Before liberation, once an individual was diagnosed with COVID-19, he and his contacts and even sub-contacts would be placed in state-enforced quarantine, where they could no longer move around freely until they were no longer contagious. After liberation, even if individuals were infected with COVID-19, they would not be forced to quarantine and could still move freely, which would promote the epidemic. Therefore, we calculated December 5, 2022 as day 0 of COVID-19 infection, December 6, 2022 as day 1 of COVID-19 infection, and so on. Considering that the peak of infection was in the second and third weeks, we finally defined the susceptible group as infected within the first four weeks, and defined the non-susceptible group as infected after the fourth week or never infected.

This study was approved by the ethics committee of Ningbo Medical Center Lihuili Hospital (Approval No: Li Huili Hospital Ethics Review 2023 Research No. 037).

### Statistical analysis

Categorical variables were reported as number (%), and the significance was tested by the chi-square or Fisher’s exact test. Abnormally distributed continuous variables were expressed as medians (interquartile ranges), and the significance was detected by the Mann–Whitney U test. A multivariate logistic regression analysis was performed by taking the susceptibility to COVID-19 (yes or no) as dependent variable and all potential confounders (age, sex, BMI, basic disease, and vaccination status) as independent variables. A *P* value less than 0.05 was considered statistically significant. All data were analyzed by SPSS statistical software, version 24.0 (IBM, Armonk, NY, USA) and GraphPad PRISM 6.0 software (GraphPad Software, San Diego, CA, USA).

## Results

A total of 870 patients who were routinely hospitalized in Ningbo Medical Center Lihuili Hospital from March 1, 2023 to March 31, 2023 were randomly selected to enroll. At the same time, in order to minimize selection bias, 28,321 Rh (D) positive hospitalized patients from the same hospital prior to the onset of the local COVID-19 outbreak throughout 2019 were selected as controls. We found no difference in ABO blood group distribution between the two groups (*P*<0.05) (Table 1).

**Table 1:** The difference in the distribution of ABO blood group between the study group and the control group.

Factor	Study group [n(%)]	Control group [n(%)]	*P* value
A	299 (34.4)	9086 (32.1)	0.155
B	215 (24.7)	7357 (26.0)	0.402
O	269 (30.9)	9337 (33.0)	0.205
AB	87 (10.0)	2541 (9.0)	0.297
Total	870 (100.0)	28321 (100.0)	

Table 2 shows the demographic and basic clinical characteristics of the 870 patients in the study group. Patients ranged in age from 2 to 94 years, with a median (interquartile range) of 58.0 (44.0, 68.0) years. 406 cases (46.7%) were male. A total of 782 cases (89.9%) were vaccinated against COVID-19, and 77.8% received three doses. 650 cases (74.7%) had been infected with COVID-19, with 157 cases (18.0%) in the second week and 252 cases (29.0%) in the third week, reaching the peak of infection.

**Table 2:** Demographics and basic clinical characteristics in the study group*.

Characteristics	The study group (n = 870)
Age (years)	58.0 (44.0, 68.0)
Gender [n (%)]	
Male	406 (46.7)
Female	464 (53.3)
BMI (kg/m^2^)	23.38 (21.02, 25.71)
Basic diseases [n (%)]	
Malignancy	236 (27.1)
Hypertension	279 (32.1)
Diabetes	115 (13.2)
Number of vaccinations [n (%)]	
0	88 (10.1)
1	16 (1.8)
2	82 (9.4)
3	677 (77.8)
4	7 (0.8)
Infection time [n (%)]	
No infection	220 (25.3)
First week	42 (4.8)
Second week	157 (18.0)
Third week	252 (29.0)
Fourth week	85 (9.8)
Fifth week or more	114 (13.1)

* Data are shown as n (%) for categorical variables and median (interquartile range) for continuous variables.

Compared with the non-susceptible group, the susceptible group had no statistically significant differences in ABO blood group and Rh phenotype, but the proportion of N+ was higher (75.6% vs 68.9%, *P* = 0.030) and the proportion of MM was lower (24.4% vs 31.1%, *P* = 0.030) (Table 3). Consistent with this, we found that ABO blood group and Rh phenotype were not significantly associated with susceptibility to COVID-19 (*P>*0.05), while N+ and MM were associated with susceptibility to COVID-19 (OR: 1.432, 95% confidence interval [CI]: 1.049, 1.954, *P* = 0.024; OR: 0.698, 95% CI: 0.512, 0.953, *P* = 0.024, respectively), after adjusting for age, sex, BMI, basic disease, and vaccination status in multivariate logistic regression analysis (Table 4).

**Table 3:** Comparison of indexes between susceptible group and non-susceptible group*.

Indexes	Susceptible group (n = 536)	Non-susceptible group (n = 334)	*P* value
Age (years)	55.0 (40.0, 67.0)	61.5 (50.0, 71.0)	<0.001
Male [n (%)]	222 (41.4)	184 (55.1)	<0.001
BMI (kg/m^2^)	23.44 (21.07, 25.78)	23.27 (20.96,25.39)	0.303
Basic diseases [n (%)]			
Malignancy	149 (27.8)	87 (26.0)	0.572
Hypertension	159 (29.7)	120 (35.9)	0.054
Diabetes	60 (11.2)	55 (16.5)	0.026
Vaccinated [n (%)]	480 (89.6)	302 (90.4)	0.680
ABO blood group [n (%)]			
A	188 (35.1)	111 (33.2)	0.578
B	121 (22.6)	94 (28.1)	0.064
O	174 (32.5)	95 (28.4)	0.212
AB	53 (9.9)	34 (10.2)	0.889
Rh phenotype [n (%)]			
antigen			
C+	477 (89.0)	301 (90.1)	0.599
c+	306 (57.1)	186 (55.7)	0.685
E+	264 (49.3)	163 (48.8)	0.897
e+	493 (92.0)	311 (93.1)	0.538
phenotype			
CCDee	224 (41.8)	143 (42.8)	0.766
CcDEe	197 (36.8)	126 (37.7)	0.773
CcDee	45 (8.4)	27 (8.1)	0.871
ccDEE	38 (7.1)	23 (6.9)	0.909
ccDEe	18 (3.4)	9 (2.7)	0.583
CCDEe	6 (1.1)	5 (1.5)	0.863
ccDee	3 (0.6)	1 (0.3)	1.000
CcDEE	5 (0.9)	0 (0.0)	0.163
MN blood group [n (%)]			
M+	401 (74.8)	253 (75.7)	0.756
N+	405 (75.6)	230 (68.9)	0.030
MM	131 (24.4)	104 (31.1)	0.030
MN	270 (50.4)	149 (44.6)	0.098
NN	135 (25.2)	81 (24.3)	0.756

The susceptible group was defined as infected within the first four weeks, and the non-susceptible group was defined as infected after the fourth week or never infected.

* Data are shown as n (%) for categorical variables and median (interquartile ranges) for continuous variables.

**Table 4:** Multivariate logistic regression analysis of ABO blood type, Rh phenotype, and MN blood type associated with susceptibility to COVID-19*.

Indexes	OR (95%CI)	*P* value
ABO blood group		
A	1.025(0.763–1.377)	0.872
B	0.764 (0.555–1.051)	0.098
O	1.247 (0.918–1.692)	0.157
AB	0.989 (0.621–1.574)	0.963
Rh phenotype		
antigen		
C+	0.824 (0.520–1.307)	0.412
c+	1.073 (0.810–1.421)	0.622
E+	1.022 (0.773–1.351)	0.878
e+	0.792 (0.463–1.357)	0.396
phenotype		
CCDee	0.946 (0.714–1.254)	0.699
CcDEe	0.932 (0.698–1.243)	0.631
CcDee	1.075 (0.647–1.788)	0.780
ccDEE	1.090 (0.629–1.889)	0.758
others	1.474 (0.773–2.809)	0.239
MN blood group		
M+	0.981 (0.710–1.358)	0.910
N+	1.432 (1.049–1.954)	0.024
MM	0.698 (0.512–0.953)	0.024
MN	1.316 (0.994–1.743)	0.055
NN	1.019 (0.737–1.409)	0.910

* Adjusted for age (≥58.0 years or <58.0 years), sex (male or female), BMI(≥23.38 kg/m^2^ or <23.38 kg/m^2^), basic disease (malignancy: yes/no; hypertension: yes/no; diabetes: yes/no), and vaccination status (yes/no).

OR = odd ratio; CI = confidence interval.

**Fig 2:**
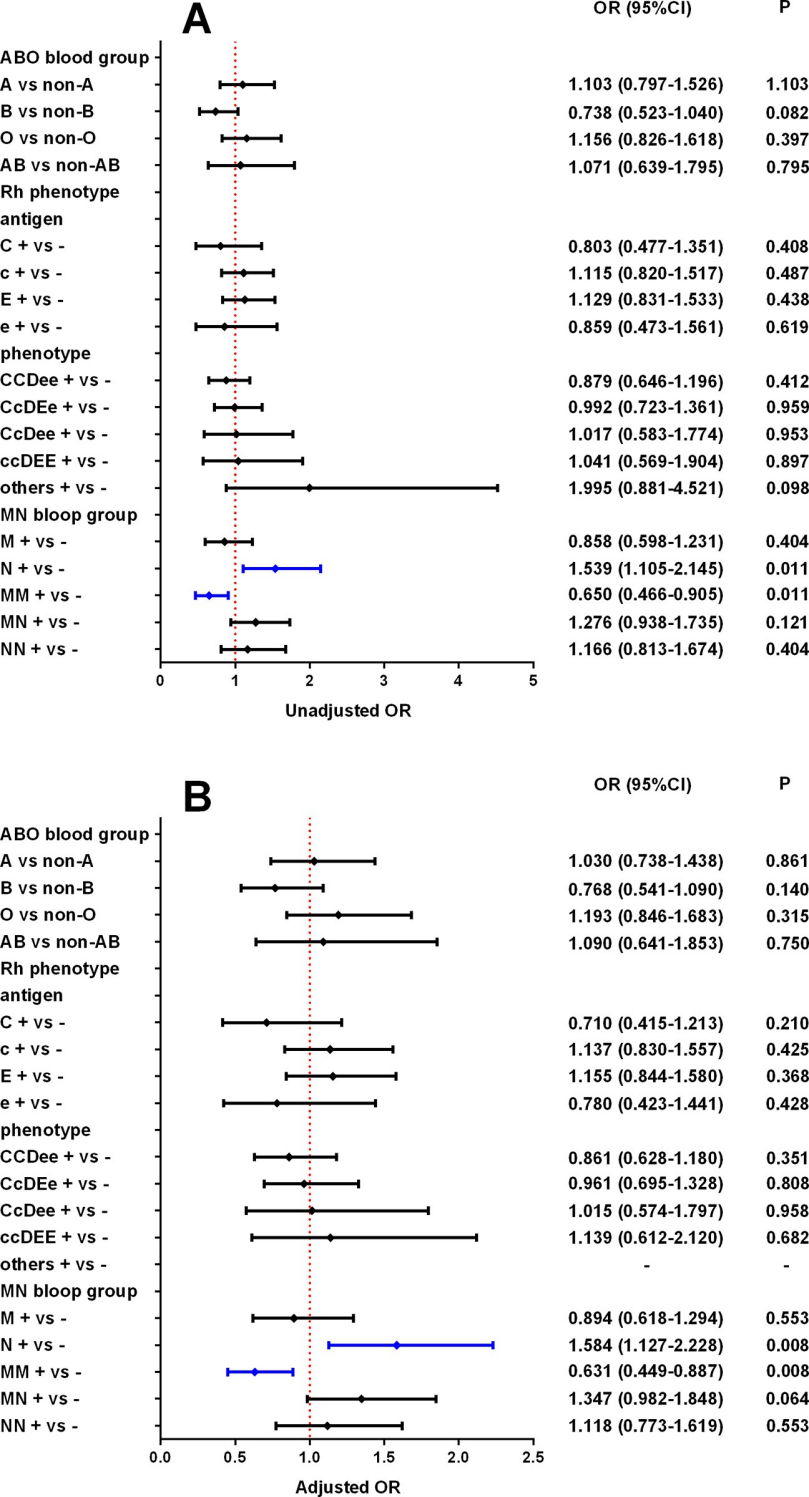
Risk of COVID-19 infection in the study population, according to ABO blood group, Rh phenotype and MN blood group. A: unadjusted; B: adjusted for age, sex, BMI, basic disease and vaccination status.

In addition, a similar phenomenon was found among all non-infected and infected people with COVID-19 (Fig 2). ABO blood group and Rh phenotype were not associated with COVID-19 infection (*P*>0.05), while N+ and MM were associated with COVID-19 infection (*P*<0.05), regardless of adjustment.

## Discussion

This prospective cohort study was performed at Ningbo Medical Center Lihuili Hospital, Ningbo University, Ningbo, China. Different from previous studies, we judged the susceptibility of COVID-19 based on the time sequence of infection. We found that ABO blood group and Rh phenotype were not associated with susceptibility to COVID-19. On the other hand, N+ was more susceptible to COVID-19 infection while MM was less susceptible to COVID-19 infection, which was an interesting discovery.

Zhao et al. [8] were the first to discover and report the correlation between ABO blood type and susceptibility to COVID-19. In their study, they found that compared with the control group, people with A blood group had a higher risk of COVID-19 infection, while those with O blood group had a lower risk. Since then, there have been more studies, many of which have found similar results [15–17]. However, no such correlation was found in this study, which is consistent with the studies of Lehrer [19], Hakami [22], etc.

In our opinion, the following reasons may be contributing to the differences. First, the study design is different. For example, in our study, the study group was all routine hospitalized patients and was grouped according to the time sequence of infection, rather than simply divided into infected and non-infected patients, which was different from other studies. Second, the differences in the distribution of ABO blood groups themselves in different regions may cause differences. Third, the confounding variables in each study were different, which may cause results bias. In addition, some studies only performed a single factor analysis, which can also cause bias.

The local population is almost all RhD positive, and RhD negative is very rare [23]. In order to reduce selection bias in Rh phenotype, three RhD negative cases were excluded and only RhD positive patients were retained. Among the four Rh antigens (C, c, E, e), the “e” antigen had the highest frequency of 92.4%, which was consistent with the results of Makroo et al [24]. In this study, CCDee was the most common Rh phenotype, accounting for 42.2%. In fact, there are a total of 9 RhD positive phenotypes, but CCDEE was not found in this study, which is very rare [24]. In addition, because several Rh phenotypes in this study had too few cases, in order to reduce bias, they were combined into other groups for statistical analysis. However, there were too few other Rh phenotypes in the non-infected group to allow for adjusted analyses of the other Rh phenotypes when comparing the infected group with the non-infected group (Fig 2). Unfortunately, we did not find an association between the Rh phenotype and susceptibility to COVID-19.

In this study, it was found that M+ accounted for 75.2% and N+ for 73.0%, which was lower in the former and higher in the latter than in the study of Halawani et al [25]. Early studies have found that MN blood group is related to plasma lipid level [26] and essential arterial hypertension in young adults [27], but whether it is related to COVID-19 infection is still unknown. Fortunately, we found that MN blood group was associated with susceptibility to COVID-19, although the reason is unknown.

At present, there is no clear standard for grouping susceptible and non-susceptible groups based on the time sequence of infection. We defined infection in the first 4 weeks as susceptible based on the peak of infection, but whether this is entirely reasonable remains to be further verified. Interestingly, in our additional analysis of the infected and non-infected groups, we found that the results were consistent with those of the susceptible and non-susceptible groups, which may further validate our grouping of susceptibility based on the time sequence of infection.

The timing of infection in the patients in this study was based on recall. However, since the interval between infection and recall was no more than 4 months in all patients, there may be bias in a relatively small number of cases. In order to reduce recall bias, we excluded 5 patients who could not recall the time of infection clearly. At the same time, the vast majority of patients had infections by week 2 or week 3; however, our group assignments were based on infection at ≤week 4 or > week 4, which also minimized biased results due to recall bias.

To the best of our knowledge, this is the first study that has really looked at the time sequence of infection. Previous studies on blood group and susceptibility to COVID-19 have mostly been comparative analyses between infected and non-infected people. In our study, we calculated the time of infection based on local community liberalization to explore the correlation between blood group and susceptibility to COVID-19.

However, there are some limitations in our study. First, there may be selection bias in this study, although comparisons were made with the historical blood group distribution of hospitalized patients prior to the COVID-19 outbreak. At the same time, our study group was all hospitalized patients rather than healthy physical examination people, although malignant tumors, hypertension and diabetes were adjusted for confounding variables. Second, we collected incomplete indicators, such as patient occupation, home isolation, etc., which may bias the results. Third, it is theoretically possible that some of the uninfected people are asymptomatic infected people, because their absence of symptoms leads them to think that they are not infected and do not undergo further testing. Fourth, the sample size in the study was relatively small and it was a single-center study. Therefore, more multicenter studies with larger sample sizes are warranted.

Despite the limitations of our study, the data presented here clearly show that the temporal sequence of COVID-19 infections during a pandemic is associated with MN blood group. The establishment of the association between COVID-19 and MN blood group may provide a theoretical basis for scientific and precise prevention and control of COVID-19, and provide a basis for the prevention and control of other virus infections in the future. The detection of MN blood group itself is relatively simple, but due to the small number of cases in this study, the significance of large-scale detection of MN still needs to be further verified.

## Conclusion

Our study showed that ABO blood group and Rh phenotype may not be related to the susceptibility to COVID-19, but MN blood group may be associated with the susceptibility to COVID-19. However, more research is still needed to further prove this hypothesis.

## Supporting information

S1 File (XLS)
